# Simulation of metabolism-based herb-drug interaction: towards safe and efficacious use of NIPRD-AM1

**Published:** 2013

**Authors:** Bulus Adzu, Kudirat Bola Mustapha, Collen Masimirembwa, Obiageri Obodozie, Rukaiyatu Abdullahi Kirim, Karniyus Shingu Gamaniel

**Affiliations:** 1 *Department of Pharmacology and Toxicology, National Institute for Pharmaceutical Research and Development, PMB 21, Abuja, Nigeria*; 2 *Department of Medicinal Chemistry and Quality Control, National Institute for Pharmaceutical Research and Development, PMB 21, Abuja, Nigeria*; 3 *African Institute of Biomedical Science and Technology (AiBST), Cnr Chinhoyi Str./Jason Moyo Ave. No. 9 at LAPF Centre, Harare, Zimbabwe*; 4 *Director General/Chief Executive Officer, National Institute for Pharmaceutical Research and Development, PMB 21, Abuja, Nigeria*

**Keywords:** CYP3A4, Herb-Drug Interactions, *Nauclea latifolia*, NIPRD-AM1

## Abstract

**Objective:** To evaluate the effect of NIPRD-AM1 on CYP3A4 in order to generate clinically significant data for its safe and efficacious use.

**Materials and Methods**: NIPRD-AM1 is a phytomedicine developed from aqueous root extracts of *Nauclea latifolia* Smith (Rubiaceae) for the treatment of uncomplicated malaria. The effect of NIPRD-AM1 on CYP3A4 was measured with and without the addition of NIPRD-AM1, by testing different concentrations of the product at 37 °C in reactive mixtures with ketoconazole (2.5 µM) as the positive control.

**Results**: Results showed a very low IC_50_ value of 0.01 mg/ml similar to that of ketoconazole (0.016 mg/ml).

**Conclusion:** Metabolic processes of NIPRD-AM1 are likely to inhibit CYP3A4, with potential implication on drugs that are CYP3A4 substrates. This is a promising approach for guidance towards the safe and efficacious use of NIPRD-AM1.

## Introduction

The use of phytomedicines (herbal products) for the treatment of diseases is well-known throughout history. A lot of them show genuine pharmacological virtues and represent a great deal of untapped reservoir of drugs. However, concerns have always been raised about their safety given the complexities of their numerous active constituents; all of which have different parameters impacting on their pharmacokinetics, especially when used in combination with synthetic drugs. Phytomedicines have been shown to cause clinically significant interaction when combined with conventional medicines (Fugh-Berman, 2000 ▶). 

This poses a risk for herb-drug interaction, thereby altering the activity of drug metabolizing enzymes, resulting in in therapeutic failure due to inhibition or induction of such metabolic enzymes. NIPRD-AM1 is a herbal medicine developed at the National Institute for Pharmaceutical Research and Development (NIPRD), Abuja, Nigeria from aqueous root extracts of *Nauclea latifolia* Smith [Rubiaceae] –Rees, Cycl. xxiv. n. 5. (IK) (International Plants Names Index, www.ipni.org) for the treatment of uncomplicated malaria. A herbarium specimen (no. 4251) was deposited at the NIPRD. Pre-clinical evaluations showed that the plant extract is safe, exhibits *in vivo* antiplasmodial efficacy against *P. berghei berghei* infected mice (Gamaniel et al., 1997 ▶), has CNS activity that is sedative in nature (Amos et al., 2005 ▶), and is efficient against experimentally induced pain, inflammation, and pyrexia (Abbah et al., 2010 ▶). The product had undergone Phase II randomized comparative clinical trials against symptomatic but uncomplicated malaria under a collaborative program with the WHO/TDR (Gamaniel, 2005). The physicochemical and other quality variables of NIPRD-AM1 have been reported (Ameh et al., 2010 ▶). In this study, we tested the effect of NIPRD-AM1 on major metabolizing enzyme, notably cytochrome P450 (CYP3A4). 

## Materials and Methods

The study was performed at the laboratories of African Institute of Biomedical Science and Technology (AiBST), Harare, Zimbabwe. Briefly, NIPRD-AM1 was ground into fine powder using pestle and mortar, and 100 mg/ml suspension was prepared in appropriate volume of 100% dimethyl sulfoxide (DMSO) and stored at -20 °C. Prior to the study, the mixture was sonicated at intervals of 30 s for 5 min using Thermo Fisher Scientific (Waltham, MA) followed by immersion of samples in ice water, then cooled back to room temperature (Gwaza et al., 2009 ▶).

Effect of NIPRD-AM1 was investigated using recombinant CYP3A4 and fluorescent-based marker reactions. It was first investigated at two concentrations calculated on the basis of original suspension [low (0.03 mg/ml) and high (0.20 mg/ml)] against human recombinant enzymes CYP3A4 (Cypex Ltd, UK) at 37 ^o^C in reactive mixtures (totaling 700 µl) consisting of 0.4 mg/ml pooled human liver microsomes (Celsis In Vitro Technologies, Baltimore), 50 mM pH 7.4 sodium/potassium phosphate buffer, 100 µM 6β-hydroxy testosterone (Sigma-Aldrich, St. Louis, MO), and 1 mM NADPH (Gwaza et al., 2009 ▶) with and without the addition of NIPRD-AM1. Ketoconazole (a known potent inhibitor of CYP3A4) (2.5 µM) was used as the positive control (Gwaza et al., 2009 ▶). The extent of activity was evaluated through serial dilutions (0.005 – 5 mg/ml). Inhibition of the CYP activity by more than 20% was considered significant and the IC_50 _(concentration of inhibitor bringing about 50% inhibition of enzyme activity) determined using non linear regression as described by Gwaza et al. (2009) ▶. 

## Results

**Figure 1:**
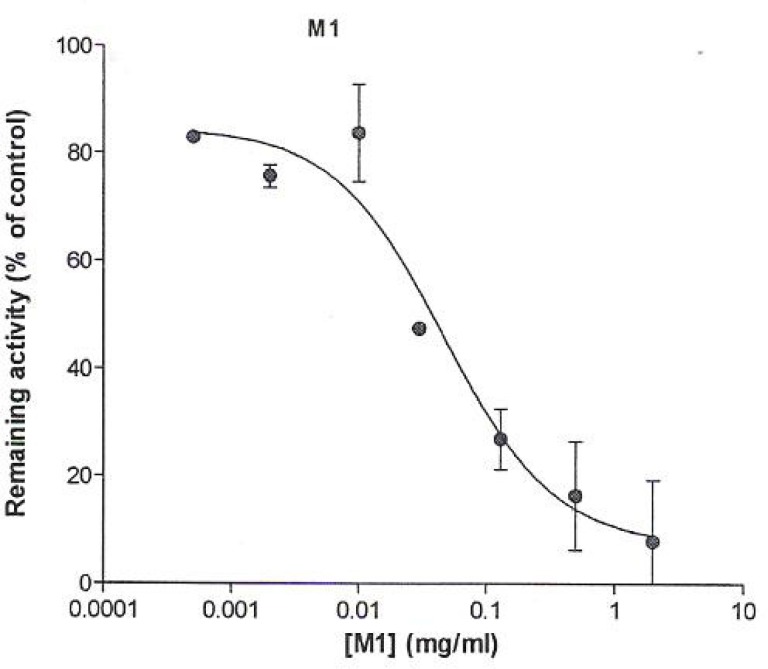
Effect of NIPRD-AM1 on CYP3A4 activity

**Figure 2:**
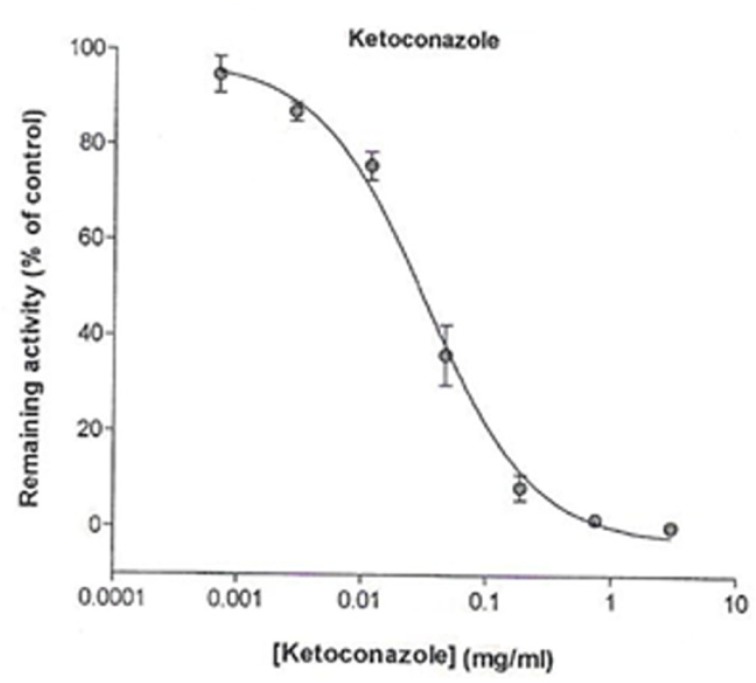
Effect of ketoconazole on CYP3A4 activity

The assay showed that NIPRD-AM1 inhibited CYP3A4 activity by more than 50%, indicating that it is a potent inhibitor of CYP3A4. It showed a very low IC_50_ value of 0.01 mg/ml similar to that of ketoconazole (0.016 mg/ml). The IC_50_ profiles are presented in Figures 1 and 2, respectively. 

## Discussion

The majority of known drugs are metabolized hepatically by mixed function oxidation reactions catalyzed by CYP 450 enzymes (Wienkers and Health, 2005 ▶), notably CYP3A4, the most important CYP isoforms in drug human metabolism (Evans and Relling, 1999 ▶; Nerbert and Russell, 2002 ▶). The interactions of a herbal product with these CYP systems may affect the fate of other drugs and their possible effects in the body. Dramatic changes in the pharmacokinetics and efficacy of herbal drugs by CYP P450 induction or inhibition have been described extensively in many studies (Monera et al., 2008 ▶; Gwaza et al., 2009 ▶).

Despite their limitations, *in vitro* assays are the most practical means of screening for potential interaction for phytomedicines (Gwaza et al., 2009 ▶). Although the observation might not necessarily result in major interaction *in vivo*, it provide useful clue for guidance towards safe and efficacious use of phytodrugs. In conclusion, NIPRD-AM1 is a potent inhibitor of CYP3A4. It should therefore be administered with caution along with CYP3A4 substrates (e.g. nifedipine, cyclosporine, erythromycin, and terfenadine). Work is ongoing in our labs to evaluate the effect of the products on the remaining important isoforms of CYP450, i.e., CYP2C9, CYP2C19, CYP1A2, and CYP2D6.

## References

[B1] Abbah J, Amos S, Chindo B, Ngazal I, Vongtau HO, Adzu B, Farida T, Odutola AA, Wambebe C, Gamaniel KS (2010). Pharmacological evidence favouring the use of Nauclea latifolia in malaria ethnopharmacy. J Ethnopharmacol.

[B2] Ameh S, Obodozie O, Gamaniel S, Abubakar M, Garba M (2010). Physicochemical variables and real time stability of the herbal substance of Niprd-AM1®-an antimalarial developed from the root of Nauclea latifolia S.M. (Rubiaceae). Int J Phytomed.

[B3] Amos S, Abbah J, Binda L, Adzu B, Buhari S, Odutola AA, Wambebe C, Gamaniel K (2005). Neuropharmacological effects of the aqueous extract of Nauclea latifolia root bark in rats and mice. J Ethnopharmacol.

[B4] Evans WE, Relling MV (1999). Pharmacogenomics: translating functional genomics into rational therapeutics. Science.

[B5] Fugh-Berman A (2000). Herb-drug interactions. The Lancet.

[B6] Gamaniel K, Wambebe C, Amupitan J, Hussaini IM, Amos S, Awodogan MO, Usman H, Enwerem N (1997). Active column fractions of Nauclea latifolia on Plasmodium berghei on rabbit ileum. J Pharm Res Dev.

[B7] Gamaniel K (2008). A comparative randomized clinical trial of NIPRD-AM1 against a chloroquine and sulphodoxine pyrimethamine combination in symptomatic but uncomplicated malaria. Abstract of the World Congress on Medicinal and Aromatic Plants.

[B8] Gwaza L, Wolfe AR, Benet LZ, Guglielmo BJ, Chagwera TE, Maponga CC, Masimirembwa CM (2009). In vitro inhibitory effects of Hypoxis obtuse and Dicoma anomala on cyp450 enzymes and p-glycoprotein. Afr J Phar Pharmacol.

[B9] Nerbert DW, Russell DW (2002). Clinical importance of the cytochromes p450. The Lancet.

[B10] Monera TG, Wolfe AR, Maponga CC, Benet LZ, Guglielmo J (2008). Moringa oleifera leaf extracts inhibit 6-hydroxylation of testosterone by CYP3A4. J Infect Dev Ctries.

[B11] Wienkers LC, Heath TG (2005). Predicting the in vivo drug interactions from in vitro drug discovery data. Nat Rev Drug Disc.

